# Heterogeneity and Breadth of Host Antibody Response to KSHV Infection Demonstrated by Systematic Analysis of the KSHV Proteome

**DOI:** 10.1371/journal.ppat.1004046

**Published:** 2014-03-27

**Authors:** Nazzarena Labo, Wendell Miley, Vickie Marshall, William Gillette, Dominic Esposito, Matthew Bess, Alexandra Turano, Thomas Uldrick, Mark N. Polizzotto, Kathleen M. Wyvill, Rachel Bagni, Robert Yarchoan, Denise Whitby

**Affiliations:** 1 Viral Oncology Section, AIDS and Cancer Virus Program, Frederick National Laboratory for Cancer Research, Frederick, Maryland, United States of America; 2 Department of Epidemiology, Johns Hopkins Bloomberg School of Public Health, Baltimore, Maryland, United States of America; 3 Protein Expression Laboratory, Advanced Technology Program, Frederick National Laboratory for Cancer Research, Frederick, Maryland, United States of America; 4 HIV and AIDS Malignancy Branch, National Cancer Institute, Bethesda, Maryland, United States of America; University of North Carolina at Chapel Hill, United States of America

## Abstract

The Kaposi sarcoma associated herpesvirus (KSHV) genome encodes more than 85 open reading frames (ORFs). Serological evaluation of KSHV infection now generally relies on reactivity to just one latent and/or one lytic protein (commonly ORF73 and K8.1). Most of the other polypeptides encoded by the virus have unknown antigenic profiles. We have systematically expressed and purified products from 72 KSHV ORFs in recombinant systems and analyzed seroreactivity in US patients with KSHV-associated malignancies, and US blood donors (low KSHV seroprevalence population). We identified several KSHV proteins (ORF38, ORF61, ORF59 and K5) that elicited significant responses in individuals with KSHV-associated diseases. In these patients, patterns of reactivity were heterogeneous; however, HIV infection appeared to be associated with breadth and intensity of serological responses. Improved antigenic characterization of additional ORFs may increase the sensitivity of serologic assays, lead to more rapid progresses in understanding immune responses to KSHV, and allow for better comprehension of the natural history of KSHV infection. To this end, we have developed a bead-based multiplex assay detecting antibodies to six KSHV antigens.

## Author Summary

Kaposi sarcoma-associated herpesvirus (KSHV) is the cause of Kaposi sarcoma, primary effusion lymphoma and a form of multicentric Castleman's disease, affecting mainly persons with HIV, other immunosuppressed patients and elderly men. Such diseases are most common where KSHV prevalence is high, in sub-Saharan Africa and the Mediterranean, and amongst men who have sex with men. Various assays for the serodiagnosis of KSHV have been developed to investigate global KSHV epidemiology. ELISAs utilizing one lytic (K8.1) and one latent antigen (ORF73) are often used. However, a more complete characterization of immune responses to all KSHV antigens may have important epidemiologic and clinical applications. We systematically expressed and purified 73 of the 85 proteins encoded by KSHV and analysed serologic responses in patients with KSHV-associated malignancies compared to healthy subjects. We identified significant reactivity to ORF38, ORF61, ORF59 and K5, in addition to the known K8.1, ORF73 and ORF65. Reactivity patterns were varied; however, HIV infected individuals were reactive to more antigens, with greater intensity. Next, we developed a bead-based assay that can test a small sample simultaneously for six KSHV antigens. The new tool can improve the detection of KSHV and the characterization of asymptomatic infection and KSHV associated diseases.

## Introduction

Kaposi sarcoma-associated herpesvirus (KSHV) is the causative agent of Kaposi sarcoma (KS), primary effusion lymphoma (PEL) and a type of multicentric Castleman's disease (MCD) [1]–[3]. Unlike other human herpesviruses, KSHV is not ubiquitous in human populations. The prevalence of KSHV infection generally parallels the incidence of KS, and varies strikingly according to geography, ethnicity, and certain behavioral risk factors [4]. Prevalence is very high in sub-Saharan Africa, ranging from 35 to 60% [4]–[7] and elevated in Mediterranean regions, from 10 to 30% [8], [9]. In South America, prevalence is high in Amerindians but not in non-Amerindians living in adjoining areas in comparable conditions [10], [11]. In the US and Western Europe, prevalence is generally low but is elevated in men who have sex with men (MSM) [12], [13] and in those born in certain areas of elevated KSHV prevalence [14].

These observations are built on more than fifteen years of sero-epidemiological studies based on various assays for detecting KSHV antibodies [15]. Commonly used tests include immunofluorescence assays (IFA) using either latent or lytic PEL cell lines [16] and ELISAs based on a small number of recombinant antigens or peptides [17]. We have developed and extensively used ELISAs based on recombinant K8.1 (a lytic gene) and ORF73 (a latent gene) [18]. More recently, luciferase immunoprecipitation system (LIPS) technology had been applied to KSHV serology with considerable success [19], [20].

While current assays are useful and reasonably reliable for understanding KSHV epidemiology, none have been specifically developed for diagnosis of KSHV infection in individual subjects, particularly asymptomatic persons [21]–[23]. In our epidemiological studies we usually classify subjects as seropositive when they are reactive to either ORF73 or K8.1. We have shown that a subject may be reactive to ORF73 for many years prior to showing reactivity to K8.1 and *vice versa*. The pattern of reactivity is not predictable [24], [25]. Longitudinal studies utilizing other assays have also demonstrated that seroreactivity to individual antigens fluctuates over time [26]–[28], suggesting that evaluation of additional KSHV antigens may increase test sensitivity.

The KSHV genome encodes more than 85 proteins, and most have yet to be studied in terms of seroreactivity. A recent study described KSHV and EBV protein arrays, but very few subjects were examined using this novel tool [29]. More sophisticated serologic assays are necessary to further investigate the natural history of KSHV infection and KSHV-associated malignancies.

In order to address these issues, we have expressed 72 KSHV ORFs and used the purified recombinant proteins to systematically screen for serological reactivity by ELISA. We tested individuals with either previous diagnoses of KSHV-associated diseases, or a low likelihood of KSHV infection. Such systematic analysis of the KSHV proteome allows a more complete investigation of serologic responses arising during asymptomatic KSHV infection and KSHV associated diseases. This will facilitate a better characterization of the natural history of KSHV infection and the immune responses to the virus, and may provide possible clues to further investigate disease pathogenesis. Improvement of KSHV serodiagnosis would be an immediately applicable outcome of this process.

## Materials and Methods

### Subjects and samples

Eighty two individuals were identified from two cohorts of subjects (Table 1). Forty-three healthy donors were chosen from amongst participants of the Research Donor Program of the Occupational Health Service at the Frederick National Laboratory for Cancer Research, Frederick, MD (RDP group). Healthy US blood donors have a low (<5%) seroprevalence of KSHV infection [30], and subjects in this group were therefore considered KSHV uninfected; moreover, all RDP subjects are HIV seronegative. Thirty nine KSHV infected subjects with pathologically confirmed KSHV-associated malignancies were selected from HIV and AIDS Malignancy Branch (HAMB) protocols at the National Cancer Institute, Bethesda, MD, including 36 HIV-infected and 3 HIV-uninfected subjects (HAMB group). The group included 25 patients with KS, 19 with a history of MCD (not symptomatic at time of evaluation), and 3 with PEL treated with immunochemotherapy; some subjects had more than one KSHV-associated malignancy. Samples were collected June 2010–March 2011 from HAMB subjects and February 2010–December 2010 from RDP subjects. All subjects were enrolled on study protocols approved by the relevant National Cancer Institute Institutional Review Board. All patients gave written informed consent in accordance with the Declaration of Helsinki.

**Table 1:** Characteristics of study participants.

Subject Characteristic (Cohort)	Number (% of Cohort) or Median (Range)
Healthy blood donors (RDP)#	43
*Age*	43 (24–64)
*Sex*	
Male	22 (51%)
Female	21 (49%)
*Race/ethnicity*	
Caucasian	34(79%)
Black	2(5%)
Asian	6(14%)
Hispanic	1(2%)
Patients with Confirmed KSHV Malignancies (HAMB)*	39
*Age*	45 (30–70)
*Sex*	
Male	35 (90%)
Female	4 (10%)
*Region of Birth* #	
North America	24 (62%)
Latin America	5 (13%)
West Africa	5 (10%)
East Africa	4 (10%)
North Africa	1 (3%)
*Race/ethnicity*	
Caucasian	19(49%)
Black	14(36%)
Unknown	5(13%)
Multiple	1(3%)
*Time since diagnosis of KSHV-malignancy (years)*	5.4 (0.4–13.7)
*Clinical characteristics by HIV status*	
*HIV-uninfected*	3 (8% of HIV-uninfected)
CD4 Count (cells/uL)†	275 (171–366)
Kaposi Sarcoma	3 (100% of HIV-uninfected)
*HIV-infected*	36 (92%)
CD4 Count (cells/uL)	458 (79–1072)
Kaposi Sarcoma	22 (61% of HIV-infected)
Multicentric Castleman's Disease	19 (53% of HIV-infected)
Primary Effusion Lymphoma	3 (8% of HIV-infected)

# For all subjects, the United States is the country of current domicile, but not necessarily the country of birth; African regions described using United Nations designations.

*Patients had Kaposi sarcoma and/or primary effusion lymphoma and/or multicentric Castleman's disease in remission (not symptomatic); some had multiple diagnoses.

† CD4 counts at time of serologic evaluation available on 33 of 36 HIV-infected subjects.

### Cloning of KSHV-encoded ORFs

#### Entry clones

Each KSHV ORF was amplified by PCR either from the KS library described in [31], obtained from Dr. Patrick Moore and Dr. Yuan Chang through the NIH AIDS Reagent Program, Division of AIDS, NIAID, NIH or from total DNA extracted from the BCBL-1 cell line (Table S1). ORFs were cloned with a C-terminal FLAG epitope tag and an N-terminal Tev protease site (ENLYFQG) followed by a Kozak translation initiation sequence. Amplification was carried out using Phusion DNA polymerase (New England Biolabs, Beverley, MA) and primers containing flanking Gateway recombination sequences, attB1 and attB2 (Life Technologies, Carlsbad, CA), under the manufacturer's standard conditions with an extension time of 30 sec/kB. Purified PCR product (QiaQuick PCR Purification Kit, Qiagen, Valencia, CA), were recombined in the Gateway Donor vector pDonr253, using a Gateway BP reaction. Entry clones were sequenced throughout the cloned regions. Spliced genes were cloned by overlap PCR in which separate amplicons with 25 bp overlapping ends were produced for each exon and fused together in a subsequent PCR. Due to amplification issues, synthetic entry clones (Life Technologies) were produced for ORF64 and ORF75.

#### Subcloning

KSHV ORF entry clones were recombined using Gateway LR into expression vectors (Table S2) for *E. coli*, baculovirus and mammalian systems. *E. coli* and baculovirus clones include a maltose binding protein (MBP) tag (His6-MBP). *E. coli* expression subclones were directly transformed into *E. coli* BL21(DE3). Baculovirus clones were converted to bacmid DNAs using the Bac-to-Bac system (Life Technologies); bacmid DNA was used to transfect insect cells. Mammalian clones were amplified, purified using GenElute XL (Sigma, St. Louis, MO), and transfected into HEK293 cells.

### Generation of recombinant antigens

#### Baculovirus-insect expression

Baculovirus stocks were prepared in Sf-9 cells grown in HyClone SFX medium and titrated using an end-point dilution assay [32].[32]. H5 cells were seeded in SFX medium at a density of 8.5×10e5 cells/ml. The culture was incubated for 24 h at 27°C and 100–110 rpms. The culture was then infected at a MOI of 3. Following incubation at 21C for 72 hrs., the supernatant was collected by centrifugation at 2000 g for 10 min.

#### *E. coli* expression

Seed cultures were inoculated from glycerol stocks (BL21* pRare) and grown in non-inducing medium (MDAG-135) for 17 hrs. at 37°C until mid-log phase growth. Buffered rich medium was then inoculated with 2% v/v of the seed culture; the cultures were grown for 4.5 hours at 37°C until the OD (A600) reached ∼5–6, chilled to 20°C, and induced with 0.5 mM IPTG. The cultures were grown for 19.5 hours; then the cells were collected by centrifugation and stored at −80°C.

#### Lysis

The cell pellets (∼20 g wet weight) were thawed and cells were resuspended in 20 mM HEPES, pH 7.3, 300 mM NaCl, 2 mM bME at ∼100 OD/ml. Protease inhibitor cocktail (P-8849 Sigma) was used at 1 ml per 10,000 ODs. Cells were lysed mechanically (three passes at 10,000 psi for E.Coli and 4,000 psi for insect cells) through a high-pressure instrument (Microfluidizer M-110EH, Microfluidics Corp, Newton, MA). Lysates were clarified by ultracentrifugation (70000 g, 30 min) and stored at −80°C until purification.

#### Purification

Clarified lysate was thawed, adjusted to 50 mM imidazole and loaded at 1 ml/min onto a 5 ml IMAC column (HisTrap, GE Healthcare). The equilibration buffer (EB) for the column was 20 mM HEPES, pH 7.3, 300 mM NaCl, 2 mM bME, 50 mM imidazole. The column was washed to baseline with EB and proteins eluted with a 20 column-volume (CV) gradient from 50 mM to 500 mM imidazole. Elution fractions were analyzed by SDS-PAGE and Coomassie-staining. Positive fractions were pooled, dialyzed to 1× PBS, pH 7.2, concentrated to ∼1.0 mg/ml, and a sub-sample tested for macroscopic stability through one freeze (−80°C)/thaw cycle before creating final aliquots for storage at −80°C. QC on final samples consisted of positive identification of at least 2 tryptic peptides by MS.

### ELISAs

The assay was carried on as previously described [18] with several modifications, as detailed below. Either plasma or serum samples were used; assay characteristics and performances had been previously determined to be equivalent in testing the two type of specimen (data not shown).

#### Plate preparation

96 well ELISA plate (Immulon 4 HBX, Dynex Technologies, Chantilly, VA) were coated with each recombinant protein at a final concentration of 0.001 µg/mL in PBS at pH 7.2 at 4°C overnight. Plates were washed and blocked in assay buffer containing 2.5% normal goat serum (Equitech-Bio, Kerrville, TX), 2.5% BSA (Sigma, St. Louis, MO), 0.005% Triton X-100, and 0.005% Tween 20 for 3 hours at 37°C. Plates were stored with assay buffer at −85°C until further use.

#### Assay procedure

ELISA plates were thawed and washed with wash buffer containing 0.05% Tween 20 in PBS, pH 7.2. Plasma or serum samples were diluted 1∶20 in assay buffer; diluted plasma (100 µL) was added to each ELISA plate and incubated at 37°C for 1.5 hours. Captured antibodies were detected using a 1∶5000 dilution of phosphatase labeled goat anti-Human IgG (H+L) antibody (KPL, Gaithersburg, MD) and 1-step PNPP substrate (Thermo Scientific, Waltham, MA); optical density (OD) was read on a Spectramax plus 384 spectrophotometer, using the program Softmax Pro v5.4.2 (Molecular Devices, Sunnyvale, CA). All steps, including preparation of master specimen plates, were performed using a STAR automated liquid handling workstation (Hamilton Robotics, Reno, NV).

### KSHV quantitative real-time PCR

KSHV-viral load was measured using previously described methods [33]. Briefly, DNA was extracted from peripheral blood mononuclear cells (PBMCs) and KSHV DNA was detected using primers for the K6 gene region. The number of cellular equivalents was determined using a quantitative assay for human endogenous retrovirus; KSHV viral load (VL) was reported as viral DNA copies per million PBMCs.

### Multiplexed bead-based assay

Antigens were covalently attached to Bio-Plex Pro Magnetic COOH beads (Biorad, Hercules, CA) via a sulfo-N-hydroxysulfosuccinimide mediated ester according to the manufacturer's protocol. To each well were added 2500 beads in 50 µL assay buffer and 50 µL of diluted serum (1∶50, 1∶100 or 1∶200), which were incubated for 1 hour at room temperature and washed. The secondary antibody, a goat F(Ab′)2 Anti-Human IgG (γ), R-PE conjugate, (Life Technologies, Grand Island, NY) was then incubated for 30 minutes; samples were washed, resuspended in 100 µL of assay buffer and analyzed on the Bio-Plex 200 system (Biorad, Hercules, CA). The median fluorescence intensity (MFI) across all counted beads was computed for each sample, and recorded after subtracting the background fluorescence.

### Statistical analysis

Seroreactivity was evaluated by comparing median ODs and MFIs using unpaired Mann-Whitney test between two groups; p-values of the U statistic were computed asymptotically (100 permutations) and Benjamini-Hochberg (B–H) corrected for multiple comparisons. For receiver operator characteristic (ROC) comparisons, the area under the curve (AUC) was computed using the trapezoidal rule, asymptotic normal p-values were Bonferroni corrected for multiple comparisons. ODs comparisons were performed using GeneSpring v11.5 (Agilent Technologies, Santa Clara, CA). All other statistical analyses were performed using Prism v6 (GraphPad, La Jolla, CA) or STATA v11.2 (Statacorp, College Station, TX).

## Results

### The KSHV proteome

Seventy three recombinant His6-MBP fusion proteins from 72 of the KSHV ORFs were expressed and purified (Table S1). The recombinant proteins included 69 expressed in baculovirus and 15 expressed in *E Coli*; 26 were expressed in both. The variable genes K1 and K15 were not cloned as part of this study. Of the 12 remaining ORFs, 4 were not successfully expressed and 8 could not be purified intact or in sufficient amount.

### Reactivity to recombinant KSHV proteins in US KSHV infected subjects and healthy donors

**Figure 1:**
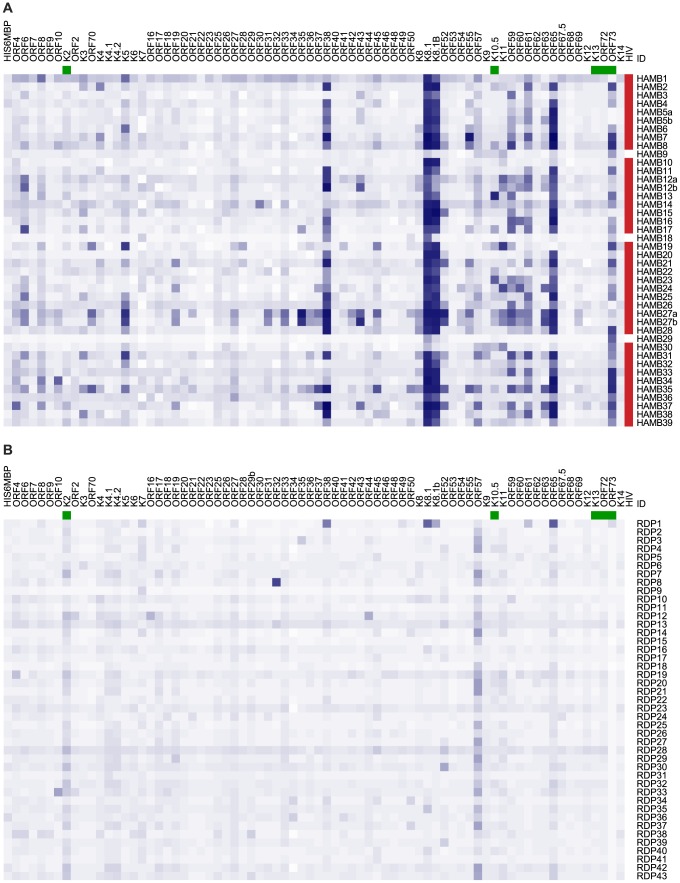
Heatmap showing seroreactivity of HAMB and RDP subjects to each antigen. A. Subjects with documented KSHV-associated diseases. B. Healthy blood donors. Subjects are shown in rows, antigens in columns. Color intensity is proportional to background-subtracted optical density (OD). HIV seropositivity is indicated in red; latent KSHV proteins are identified in green.

To assess the antigenicity of the recombinant proteins generated, we measured antibody responses by ELISA in a panel of specimens from our KSHV-infected subjects (HAMB) and healthy blood donors from the Frederick National Laboratory for Cancer Research (FNLCR) Research Donor Program donor (RDP) groups. Subject characteristics are noted in Table 1. Seroreactivity against KSHV encoded antigens are presented as a heatmap in Figure 1, where each recombinant protein is shown in a column and each subject in a row. The optical density (OD) is represented by the colour intensity. Panel A shows reactivity in subjects with documented KSHV disease (HAMB subjects) and panel B shows reactivity in healthy donors (RDP subjects). Intensity and breadth of reactivity was substantially greater in samples from HAMB subjects. One of the healthy donors (RDP1) showed reactivity to K8.1 and ORF73, as well as to 3 other antigens, consistent with KSHV infection. This observation is in accordance with estimates of KSHV prevalence in US blood donors [30].

**Figure 2:**
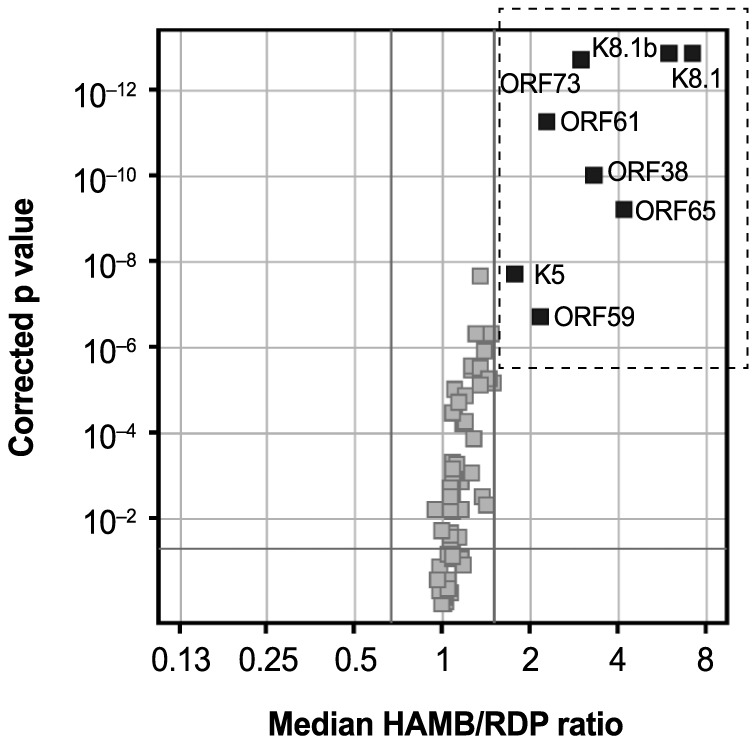
Volcano plot displaying antigens eliciting significantly different reactivity in HAMB and RDP subjects. On the horizontal axis, median HAMB/RDP reactivity ratio is indicated; the corresponding Benjamini-Hochberg (B–H) corrected p-value is shown on the vertical axis. The horizontal line represent a corrected p-value<0.05; the rightmost vertical line represents a ratio = 1.5. Thus, symbols within the dashed rectangle indicate ratios greater than 1.5 with p-value below 0.05.

To determine which responses differentiated infected and uninfected individuals, the ratio of median reactivity in HAMB and RDP subjects was calculated for each antigen. A comparison between median ODs in HAMB vs. RDP samples is shown as a volcano plot in Figure 2. The values obtained are indicated on the horizontal axis, whereas the Benjamini-Hochberg (B–H) corrected p-value of the test statistic for the difference in median reactivity between the two groups is shown on the vertical axis. Eight proteins showed a median OD ratio greater than 1.5 with a corrected p-value lower than 0.05. These were ORF73 and K8.1 (and the isoform K8.1B), ORF65, ORF38, ORF61, ORF59 and K5.

The pattern of seroreactivity observed in subjects with documented KSHV disease was highly variable. Subjects showed reactivity to as few as 2 to greater than 15 antigens. The intensity of the responses, as indicated by OD measurements, was also variable for individual antigens and subjects. Using individual OD measurements, we constructed variables summarizing the breath and the intensity of the serologic reactivity in each HAMB subject. For each antigen, OD values greater than those in the upper 5% of the distribution in the RDP donors were considered indicative of a significant reactivity. The number of such responses, and a measure of average reactivity (mean OD) was recorded for each subject. As expected from previous reports, responses to K8.1, ORF73, and ORF65, were observed in a majority of HAMB subjects (81–100%); responses to K8.1 and to the K8.1B splice variant [34] were quite similar. Reactivity to ORF38, ORF61 ORF59 and K5 was also observed in a high proportion of subjects (74–86%). Some proteins, like ORF23 and ORF32, did not appear to be antigenic in this system, or in these HAMB subjects, and ORF57 and K2 appeared to elicit responses in both HAMB and RDP subjects, suggesting that such reactivity is non-specific.

To explore the association between serologic responses and KSHV DNA detection in patients with a history of KSHV associated malignancies, KSHV viral load (VL) in peripheral blood mononuclear cells (PBMCs) was determined in HAMB subjects at the time of sampling for serology; 7 (26%) had detectable KSHV VL. Additionally, in 35 of the subjects, KSHV VL had also been measured longitudinally prior to sampling. The number of measurements varied between 3 and 25 (mean 7.7, Standard Deviation 6.12) and the length of retrospective follow up varied between 77 days and 13 years (mean 1615, SD 1356 days). Thirty-three subjects (87%) had detectable KSHV during at least one visit. Neither detectable KSHV VL at the time of serum collection nor history of KSHV VL (expressed as mean VL over the entire follow up, peak VL, or ever-positive VL) were associated with the breadth or intensity of responses. The same was observed when individual KSHV VL measurements were introduced in a longitudinal model.

Results did not change when the analyses were restricted to lytic antigens, or after dropping the 3 PEL patients, who were receiving immunochemotherapy potentially affecting antibody response. . In contrast, history of HIV infection appeared to affect reactivity in subjects with documented KSHV-associated malignancies. Three HAMB subjects in this panel were HIV negative (noted in Figure 1); they showed reactivity to fewer antigens (5.3, 95%CI 0–11.6, vs. 26.6, 95%CI 21.1–32.14) and with lower mean ODs (0.2, 95%CI 0.0–0.4, vs. 0.6, 95%CI 0.5–0.7).

### Development of a multiplex KSHV serological assay

In order to build upon the findings of our screening assays in a practical, translational manner, we next developed a multiplex bead-based assay that allows concurrent testing of the six antigens that demonstrated median OD ratio between RDP and HAMB subjects greater than 2: K8.1, ORF73, ORF65, ORF38, ORF59 and ORF61. K8.1B was not included because K8.1 and K8.1B provided nearly identical data. The assay requires only one microliter of serum, and this approach has been successful for other pathogens in both epidemiological and diagnostic settings [35], [36].

**Figure 3:**
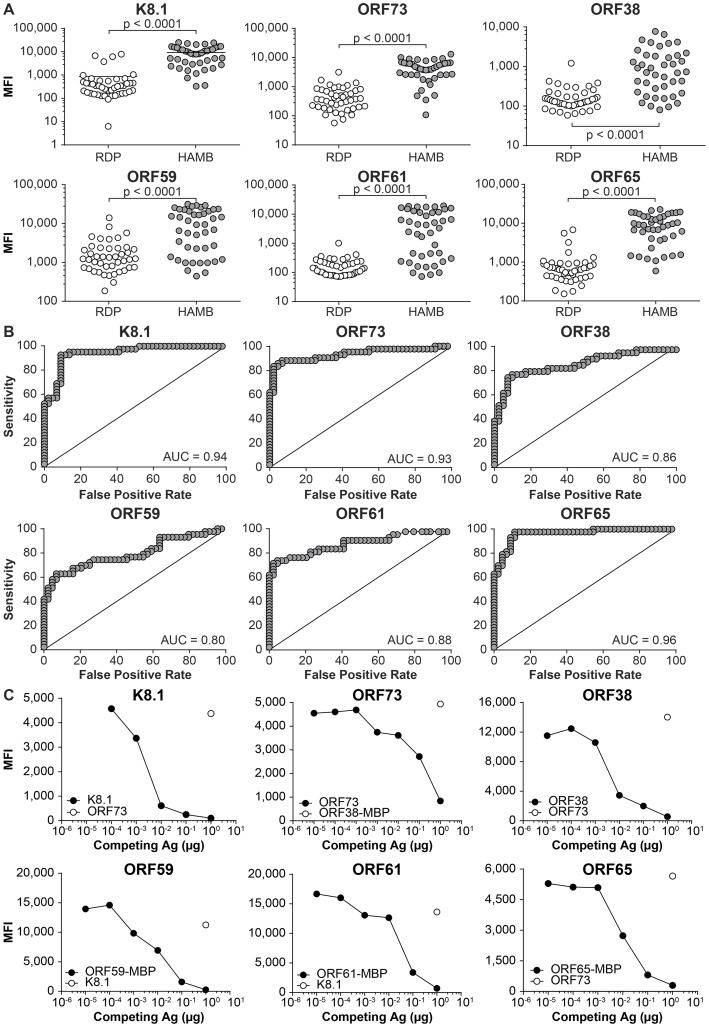
Bead-based assay. **A**. Comparison of reactivity to individual antigens in RDP and HAMB subjects (Mann-Whitney test) **B**. Receiver operating characteristics. **C**. Assessment of signal specificity by antigen pre-adsorption. MFI, Median Fluorescence Intensity.

Single-plex and then multiplex assay parameters were optimized (data not shown). HAMB and RDP samples were re-analyzed in multiplex for the 6 antigens. In Figure 3, panel A shows the distributions of MFI values in the HAMB and RDP group for each antigen. Differences in reactivity between RDP and HAMB subjects were confirmed to be statistically significant using the multiplex assay (corrected p-value less than 0.0001 for each comparison). Panel B displays the receiver operating characteristics [37] (ROC) for each of the antigens, demonstrating that all assays discriminate between KSHV infected subjects and healthy donors. Sensitivity and specificity were 87% and 95% for K8.1, 92% and 95% (ORF73), 87% and 82% (ORF65), 72% and 95% (ORF38), 69% and 86% (ORF61), 62% and 95% (ORF59). The single KSHV infected healthy donor was retained in the RDP group for all analyses. Had the individual been excluded or included in the HAMB group, sensitivity and specificity estimates would have not changed appreciably.

**Figure 4:**
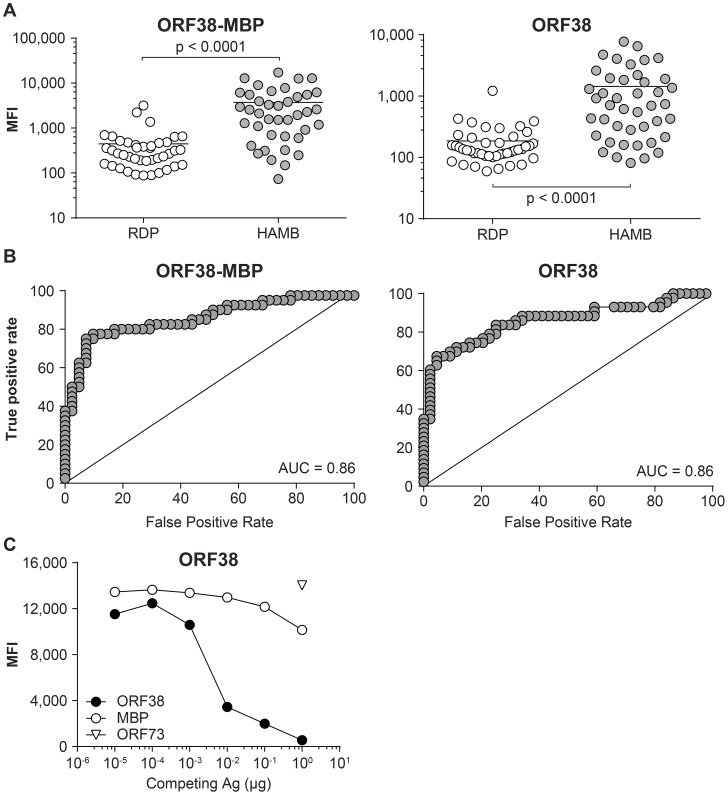
Bead-based assay. Assessment of non-specific reactivity to purification solubility tag Maltose Binding Protein (MBP) **A**. Comparison of reactivity to individual antigens in RDP and HAMB subjects (Mann-Whitney test) **B**. Receiver operating characteristics. **C**. Assessment of signal specificity by antigen pre-adsorption. MFI, Median Fluorescence Intensity.

Analytic specificity was demonstrated by pretreating samples with the appropriate antigen or an irrelevant antigen, prior to adding antigen-conjugated beads. Specific IgG antibodies in serum bind to the soluble antigen, preventing adsorption to the antigen-conjugated beads and leading to a loss of signal (Panel C). In order to determine if the signal was due to non-specific binding to the MBP purification solubility tag fused to the antigen, reactivity to ORF38 either fused to MBP or cleaved from MBP was measured; the two were shown to be equivalent (Figure 4). Analytical specificity testing, performed as detailed above, further indicated that seroreactivity derived from the specific portion of the protein, not from the solubility tag.

### Comparison between bead-based assay format and ELISA

**Figure 5:**
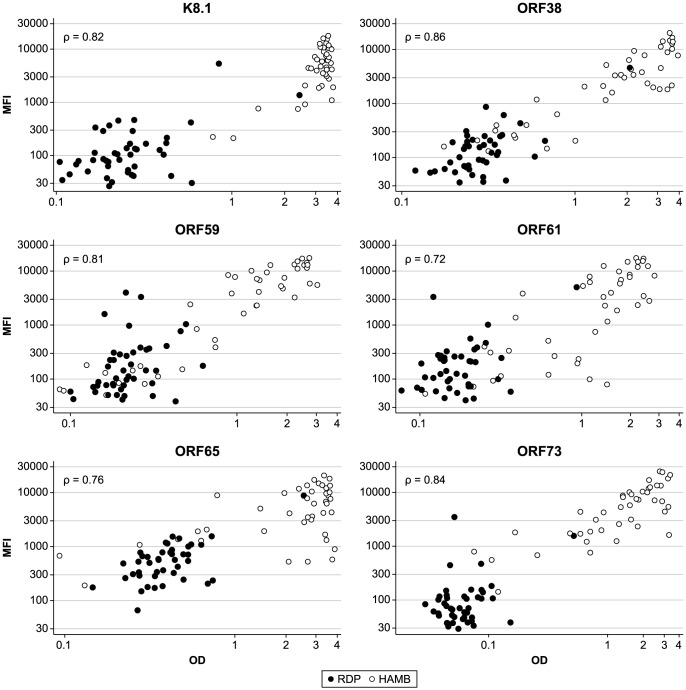
Correlation between MFI in the bead based multiplex assay and OD in single antigen ELISAs. ρ, Spearman's rank correlation coefficient. OD, Optical Density; MFI, Median Fluorescence Intensity.

Having ascertained that the sensitivity and specificity of the newly developed multiplex assay for each of the 6 KSHV antigens were satisfactory, we examined its quantitative features compared to those of individual ELISAs. Specifically, we evaluated the dynamic range of the new assay format, as the relatively narrow dynamic range of ELISA limits the use of ODs as a marker of antibody levels. The MFIs recorded for each antigen in the bead-based assay were plotted against the ODs obtained in the corresponding ELISA (Figure 5). For each antigen, the two sets of measurement correlated well (Spearman's rank coefficient varied between 0.76 and 0.86, p<0.00001 for all comparisons).The ELISA generally presented a smaller dynamic range, here defined as the ratio between the largest and smallest detected values (mean, 61; SD, 59 vs. mean, 560; SD, 197), which compresses the highest OD values. The difference is particularly evident for reactivity to KSHV capsid proteins K8.1 and ORF65. These findings did not vary when the bead-based assay was repeated using a different serum dilution, confirming the quantitative robustness of the assay. These data demonstrate the greater dynamic range of the bead-based assay format, compared to ELISA, which is likely to facilitate semi-quantitative analysis of antibody data, an important feature of this assay that will be useful for future KSHV research.

## Discussion

In order to systematically evaluate the antigenic characteristics of KSHV-encoded proteins, we sought to express the entire KSHV proteome. We obtained recombinant proteins from 72 of the more than 85 described ORFs. The K1 and K15 genes were excluded from this study because of their high variability. Expression and/or purification of the few remaining proteins were not possible due to various technical issues.

The initial ELISA screening of these recombinant proteins with a panel of well characterized subjects with KSHV-related disease and healthy donors revealed a defined pattern of reactivity. A low background was detected in the healthy donors, as compared to strong and diverse reactivity in the KSHV infected subjects. Seven antigens appeared to strongly discriminate the two populations. These were the previously described ORF73, K8.1 (including the splice variant K8.1b) and ORF65, and the newly identified ORF38, ORF61, ORF59 and K5. However, individual patients showed different response patterns to the various antigens. One of the 44 healthy donors, who was seroreactive to K8.1, ORF73, ORF65 as well as to ORF38 and ORF61, was deemed to be KSHV infected. KSHV seroprevalence in the RDP group as determined by this assay was 2.3%, consistent with published estimates for US blood donor populations [30]. However, source population and selection criteria for RDP subjects differ somewhat from those of transfusion products donors, and our convenience sample cannot be considered representative of any population.

We demonstrate a diversity of antibody responses in subjects with documented KSHV-associated malignancies, and these findings are similar to what is observed when examining immune responses to other herpesviruses [38], [39], but the reasons for such diversity are not clear. It can be hypothesized that diverse responses to KSHV antigens may result from the variability in the natural history of the infection. Important factors may include age of primary infection, frequency of reactivation and level of viral replication, co-infection with HIV or other pathogens, immune status and development of particular KSHV-associated diseases. Our sample size and composition were not designed to allow us to ascertain an association between particular patterns of seroreactivity and factors such as age, gender, diagnosis of specific KSHV malignancies or disease duration. A previous study using luceriferase immunoprecipitation systems has shown different patterns of reactivity to a lytic antigen (K8.1) and latent antigens (LANA and v-Cyclin) in patients with KS as compared to patients with MCD [20], and it will be of interest to see how the multiplex assay discriminates among different KSHV disease states. Prior or concurrent KSHV viral load did not show an association with breadth or strength of antibody reactivity in this study, in contrast with what observed in other investigations, but, again, our sample was relatively small and not intended to detect such. Further evaluation of specifically designed study samples is required. Only a few HIV negative individuals were included amongst our KSHV infected subjects. Although we were able to observe an association between HIV infection and broader, stronger responses to KSHV antigens, we remain cautious regarding the generalizability of such finding. Further studies examining the clinico-pathological correlates of the diversity and intensity of antibody responses to KSHV are warranted. In particular, it will be necessary to consider both HIV infected and uninfected populations from areas of low or high endemicity, and to investigate subjects with KSHV associated diseases, as well as asymptomatic individuals. More generally, it is imperative that findings uncovered in the present discovery cohort be externally validated on independent cohorts.

It is worth at this point considering the KSHV antigens that we have newly found to discriminate infected from non-infected persons and their role in the KSHV life cycle. The newly described antigens include several interesting KSHV-encoded proteins. ORF38 is a poorly characterized tegument protein. Serological responses against ORF38 were recently observed in a handful of AIDS-KS patients in a study utilizing a protein array platform [29]. ORF61 encodes the large subunit of the KSHV ribonucleotide reductase, which catalyzes the conversion of ribonucleoside diphosphates to the corresponding deoxyribonucleotides, controlling the cellular concentration of the latter. The ORF59 protein (PF-8), which is transcribed from the same polycistronic transcript as the ORF61 protein, is a processivity factor for the viral DNA polymerase [40]. Both are likely to be critical for viral replication. Evidence for seroreactivity to ORF59 had been identified in AIDS-KS patients in a small study [41] but no information is available on antibodies against ORF61. K5 encodes a RING-CH E3 ubiquitin ligase which downregulates human tetherin expression on the cell surfaces and targets it for endosomal degradation, thereby promoting the release of viral progeny from infected cells [42]; furthermore, it is involved in multiple immune modulation mechanisms [43]–[46]. Although its role in KSHV lytic infection and transmission is likely to be significant, its antigenic properties had not been thus far described. Recent studies of immune responses in herpesvirus infections have consistently identified significant antigens that are orthologs of ORF38, ORF61 and ORF59. These include UL39 in HSV1 [39] and HSV2 [47], UL45 in HCMV [38], as well as BORF2 and BMFR1 in EBV [29]. Further investigations on the humoral and cell mediated responses to these antigens is warranted, to determine their significance in in the natural history of herpesvirus infection and pathogenesis of associated diseases.

To this end, we developed and validated a multiplex bead based assay, which can test simultaneously for reactivity to 6 KSHV antigens with performances comparable to those obtained using individual ELISAs. However, bead based assays have a number of advantages over ELISAs. The dynamic range is wider, reducing the need for serial dilution of samples with very high antibody levels. Moreover, bead based assays can be developed to allow for semi-quantitative determination of antibody levels [48] in lieu of determining antibody titers. This is a significant characteristic, since antibody titers have been shown to be related to KSHV replication [49] and to be associated with the development of KS [50], [51]. Finally, the technology requires a much smaller sample volume, and the cost per antigen, per sample, is lower. Additional antigens can be further multiplexed into the assay as needed for specific epidemiologic studies.

In summary, this study provides a systematic antigenic analysis of the KSHV proteome. Several new antigens have been identified and relevant assays have been developed as a tool that can be evaluated for serodiagnosis and that will be useful in epidemiologic studies and characterization of KSHV associated conditions. Furthermore, cloning, expression and transduction vectors as well as recombinant proteins have been produced, which will constitute a significant resource for the KSHV research community.

## Supporting Information

Table S1 KSHV ORF clones, purified protein, and transfected cell lines produced in connection with this study.
(XLSX)

Table S2 Gateway destination clones utilized and their characteristics.
(DOCX)
