# Supplementary material for: Heterogeneity and Breadth of Host Antibody Response to KSHV Infection Demonstrated by Systematic Analysis of the KSHV Proteome
Source: PLoS Pathog. 2014 Mar 27;10(3):e1004046. doi: 10.1371/journal.ppat.1004046 (PMC3968157; doi:10.1371/journal.ppat.1004046)
Supplement: Table S2 — Gateway destination clones utilized and their characteristics. (DOCX) [file ppat.1004046.s002.docx]

**Table S2. Cloning and expression vectors.**

| **Name** | **Expression system** | **Original vector** | **Promoter** | **Tags** |
| --- | --- | --- | --- | --- |
| pDest-566 | E.Coli | pMalc2* | T7 | His6-MBP |
| pDest-636 | baculovirus | pFastBac^#^ | Polyhedrin | His6-MBP |
| pDest-780 | mammalian | pcDNA3.1^#^ | CMV | none |

* New England Biolabs

^#^ Life Technologies
